# Occupational risks associated with chronic kidney disease of non-traditional origin (CKDnt) in Brazil: it is time to dig deeper into a neglected problem

**DOI:** 10.1590/2175-8239-JBN-2023-0123en

**Published:** 2024-04-05

**Authors:** Rafael Junqueira Buralli, Polianna L M Moreira Albuquerque, Cintia da Espiritu Santo, Viviane Calice-Silva, Fabiana Baggio Nerbass

**Affiliations:** 1 Universidade de São Paulo, Faculdade de Medicina, Departamento de Medicina Preventiva, São Paulo, SP, Brazil.; 2 Universidade de Fortaleza, Faculdade de Medicina, Fortaleza, CE, Brazil.; 3 Fundação Pró-Rim, Joinville, SC, Brazil.

**Keywords:** Chronic Kidney Diseases of Uncertain Etiology, CKDnt, CKDu, Occupational Risks, Heat-Shock Response, Pesticides, Doenças Renais Crônicas Idiopáticas, DRCnt, DRCd, Riscos Ocupacionais, Resposta ao Choque Térmico, Praguicidas

## Abstract

In the past decades, an epidemic of chronic kidney disease (CKD) has been associated with environmental and occupational factors (heat stress from high workloads in hot temperatures and exposure to chemicals, such as pesticides and metals), which has been termed CKD of non-traditional origin (CKDnt). This descriptive review aims to present recent evidence about heat stress, pesticides, and metals as possible causes of CKDnt and provide an overview of the related Brazilian regulation, enforcement, and health surveillance strategies. Brazilian workers are commonly exposed to extreme heat conditions and other CKDnt risk factors, including increasing exposure to pesticides and metals. Furthermore, there is a lack of adequate regulation (and enforcement), public policies, and strategies to protect the kidney health of workers, considering the main risk factors. CKDnt is likely to be a significant cause of CKD in Brazil, since CKD’s etiology is unknown in many patients and several conditions for its development are present in the country. Further epidemiological studies may be conducted to explore causal associations and estimate the impact of heat, pesticides, and metals on CKDnt in Brazil. Moreover, public policies should prioritize reducing workers´ exposure and promoting their health and safety.

## Resumo

Nas últimas décadas, uma epidemia de doença renal crônica (DRC) tem sido associada a fatores ambientais e ocupacionais (estresse térmico decorrente de cargas de trabalho elevadas em altas temperaturas e exposição a produtos químicos, como agrotóxicos e metais), denominada DRC de origem não tradicional (DRCnt). Esta revisão descritiva tem como objetivo apresentar evidências recentes sobre estresse térmico, agrotóxicos e metais como possíveis causas de DRCnt e fornecer uma visão geral das estratégias brasileiras de regulamentação, fiscalização e vigilância sanitária relacionadas. Os trabalhadores brasileiros são comumente expostos a condições extremas de calor e outros fatores de risco de DRCnt, incluindo o aumento da exposição a agrotóxicos e metais. Além disso, há uma falta de regulamentação e fiscalização, políticas públicas e estratégias adequadas para proteger a saúde renal dos trabalhadores em relação aos principais fatores de risco. É provável que a DRCnt seja uma causa significativa de DRC no Brasil, uma vez que a etiologia da doença é desconhecida em muitos pacientes e diversas condições para seu desenvolvimento estão presentes no país. Estudos epidemiológicos devem ser realizados para explorar associações causais e estimar o impacto do calor, dos agrotóxicos e dos metais na DRCnt no Brasil. Além disso, as políticas públicas devem priorizar a redução da exposição dos trabalhadores e a promoção de sua saúde e segurança.

## Introduction

Chronic kidney disease (CKD) has a high prevalence worldwide, generating high costs for healthcare systems and high morbidity and mortality for patients, especially when the disease progresses to its more advanced stages. It is estimated that the worldwide prevalence of CKD ranges between 8–16% in the adult population^1^. In the past decades, environmental and occupational factors have been associated with CKD, especially in CKD hotspots, which are defined as countries, regions, or ethnicities with a higher than average incidence of the disease^2^.

CKD of non-traditional factors (CKDnt), also named CKD of unknown origin (CKDu) is a diagnosis of exclusion for CKD, made when a patient fulfills the Kidney Disease Improving Global Outcomes (KDIGO) CKD criteria without evidence of a recognized cause such as diabetes, hypertension, genetic disease, or glomerulonephritis^3^. It is a primarily tubulointerstitial process with nonproteinuric loss of kidney function. Patients with CKDnt are younger than patients with traditional CKD and usually work in specific occupations in certain hotspots of the world such as Central America, India, and Sri Lanka, where it has been responsible for thousands of deaths^4,5^.

Many potential etiologies of CKDnt have been studied, and it appears that exposure to heat, nephrotoxic metals, and pesticides are responsible for a great proportion of cases globally, especially among workers^6^. Heat stress and dehydration are presently the biggest focus of research in Latin America, while in Asia, drinking water contamination has been hypothesized as the primary cause of CKDnt^7^. Moreover, epidemiological studies have found higher prevalence of CKDnt among agricultural workers, such as sugarcane workers in Central America and rice farmers in Asia^8,9^.

In Brazil, it is very likely that CKDnt is a relevant cause of CKD, because the general population, especially workers, are exposed to extreme heat conditions – which is increasing due to climate change – as well as pesticides and metals^10–13^. Furthermore, the CKD etiology of many patients is unknown, according to nephrology surveys. The Brazilian Dialysis Survey, which gathers information on patients under dialysis treatment of about 30–40% of the country’s dialysis centers, reported that in the last five years, the primary cause of CKD was unknown for 10–11% of patients^14^.

Although Brazil does not have large populational-based studies, the National Health Survey (2014–2015) found an overall prevalence of estimated glomerular filtration rate (eGFR) <60 mL/min/1.73 m^2^ of 6.7% (95%CI 6.0 – 7.4) in 7,457 adults.^15^ However, albuminuria was not included in the CKD classification criteria, which may have underestimated the prevalence. Moreover, the ELSA study with public sector employees aged between 35 and 74 years found a CKD prevalence of about 9%^16^.

Furthermore, the lack of proper regulation and enforcement related to CKDnt risk factors associated with the lack of public policies and strategies to detect and treat CKD, may increase the risk of workers developing the disease. In this context, this descriptive review based on a non-systematic review of relevant studies, Brazilian regulation, and technical publications, presents recent evidence about heat, pesticides, and metals as possible causes of CKDnt and provides an overview of the related Brazilian regulation, enforcement, and health surveillance strategies. This knowledge may be useful in raising awareness of this important public health problem, promote public policies and strategies to eliminate or reduce the exposure of worker, and strengthen health services to deal with the disease.

## Occupational Risk Factors for CKDnt

Three of the main recognized occupational risk factors for CKDnt, namely heat stress, exposure to pesticides and exposure to metals, are discussed below, followed by an overview of the related regulation in Brazil.

### Heat Stress

Heat stress is the sum of the heat generated in the body (metabolic heat) and the heat from the environment (environmental heat) minus the heat lost from the body to the environment^17^. Heat stress occurs when sweat evaporation is insufficient, and other physiological changes cannot prevent the core body temperature from rising. This heat-related occupational illness is often associated with dehydration and manifests itself in a variety of symptoms, affecting the productivity^17^. Occupational heat exposure affects outdoor workers directly exposed to the sun and indoor workers who perform activities in hot environments, such as near boilers, furnaces, and ovens^18^.

The heat stress hypothesis related to kidney function is based on evidence that repeated episodes in combination with loss of water and solutes due to high temperatures, strenuous work, insufficient rehydration, and impaired heat dissipation can lead to repeated episodes of subclinical ischemic kidney injury, which over time may cause permanent kidney damage and CKD^19^. Many researchers believe that this is central to the pathophysiology of CKDnt, which occurs mainly in sugarcane workers from Central America, where it has been referred to as Mesoamerican Nephropathy^20^. However, the extent to which heat stress causes CKD, either directly or in combination with other occupational factors remains uncertain. Biological, socioeconomic, and climate factors may also interact to increase the risk of CKDnt^21^.

Heat stress effects on kidney function has been poorly studied in Brazil. In 2015, a small study (N = 28) assessed the acute effects of harvesting burnt sugarcane on renal function and found that eGFR decreased by 20% at the end of the daily work shift, and 18.5% of participants had serum creatinine rise consistent with acute kidney injury^22^. In a pilot study that included factory workers exposed (and not exposed) to heat stress, it was found that the exposed workers had a greater decline in creatinine-based eGFR during the work shift^23^. However, after 2 years of follow-up, kidney function was maintained^24^.

Although the effect of heat stress on kidney function has not been well explored in Brazil, there are considerable chances that it affects millions of workers. The threat of excessive occupational heat exposure and its consequences is particularly high in tropical countries with low-to-middle-income where large informal sector workers exist, often operating in hot, densely populated environments with high physical workloads and scant safety regulations^25^. Brazil has both tropical and subtropical climates, so it is susceptible to atmospheric situations favorable to extreme heat. It is projected that Brazil will face aggravated heat stress conditions by the end of this century as the effects of extreme warming materialize^26^.

### Metals

Metals are chemical elements with a high atomic weight and a density greater than 5 g/cm^3^ that come from many sources, such as industry, mining, and agriculture^27^. Among other health consequences, human exposure to metals by contaminated water, food intake, and air pollution may lead to kidney injury^28^. Numerous studies about endemic CKDnt have focused on occupational and environmental risk factors, but some gaps remain^29^. In Sri-Lanka studies, metals such as fluoride (F^-^), cadmium (Cd), magnesium (Mg^+^), lead (Pb), arsenic (As), and chromium (Cr) have been considered a plausible etiology for CKDnt^30^. The proximal tubular uptake of these metals from the blood flow leads to kidney inflammation, ischemia, and tubulointerstitial damage; however, the pathophysiological mechanisms of toxicity are complex and not fully understood^31^.

The gradual accumulation of fluoride from drinking water, particularly with high levels of Mg+ and Cd, have been considered an important cause of CKDnt^30^. In Thailand, female farmers of older age and living in rural areas seem to be at higher risk for CKDnt, possibly as a consequence of exposure to metals such as zinc and Cd from waste, groundwater, and well water contamination by chemical fertilizers^32^. A study conducted in Guatemala with sugarcane workers revealed that these individuals are exposed to high concentrations of metals (such as aluminum and calcium), which should be investigated in depth as an etiologic factor for CKD in this population^33^.

The measurement of metals in urine and blood samples from patients and individuals exposed to the possible risk factors may point out the etiology of the CKDnt. A cross-sectional study in a rural area in Bangladesh revealed significantly higher levels of Pb, Cd, and Cr in urine samples of CKD patients. Only confirmed cases of CKD, who did not have high blood pressure or diabetes but had abnormal levels of eGFR were recruited^34^. In Taiwan, it was observed that individuals with high levels of blood Pb and urine copper (Cu) had proteinuria and eGFR of <60 mL/min/1.73 m^2^, and high levels of urinary nickel (Ni), manganese (Mn), and Cd were significantly associated with proteinuria. Moreover, a synergistic effect of urinary Cd and Cu on proteinuria was also observed^35^.

The use of non-traditional kidney biomarkers could reveal early damage from metals, which deserves further studies. The measurement of *N*-acetyl-β-d-glucosaminidase (NAG), retinol-binding protein, and α-1-microglobulin in urine from people living in a Cd-contaminated area has indicated a possible dose-response relationship^28^. Otherwise, some studies have shown that CKD is associated with higher blood lead and cadmium levels, which could request further efforts to protect patients from these potential risks, particularly among vulnerable populations and workers^36,37^.

### Pesticides

Brazil is one of the world’s leading agricultural exporters. However, the increase in its production has been driven by a significant increase in pesticide use, making the country one of the world’s largest consumers, responsible for about 20% of the global market^38^. In 2018, approximately 550,000 tons of pesticide active ingredients were sold in Brazil^39^, being mostly herbicides (62%), fungicides (13%), and insecticides (10%)^39^. About 72% is used in agricultural business for the production of commodities such as soy, sugar cane and corn, and the rest is employed in medium and smallhodler family farming^38^. The chemical industry takes advantage of the permission of Brazilian authorities to sell their most dangerous products in the country and pesticide approvals have recently increased (as well as poisoning cases). About 30% of the pesticides sold in Brazil are banned in the European Union due to their high toxicity^38^.

Short- and long-term exposure to several pesticides commonly used in Brazil was associated with kidney effects in experimental and epidemiologic studies, including glyphosate, paraquat, chlorpyrifos, malathion, atrazine, permethrin, other OPs and pyrethroids^39,40^. The mechanisms of action of pesticides’ nephrotoxicity are not fully understood, but experimental studies have shown how different classes of pesticide can affect kidney function. Round-up, a glyphosate-based herbicide, leads to epigenetic and pathophysiological changes in kidney function^41^; pyrethroid pesticides exposure induces oxidative stress and tissue damage^42^; organophosphates pesticides (OP) trigger oxidative stress and reduce glutathione metabolism^43^; malathion, an OP insecticide, was found to cause metabolic, histopathologic and molecular disorders in liver and kidney^44^; and chlorpyrifos exposure disrupts plasma membranes, leading to tissue damage and loss of enzyme activity^45^.

Work-related exposure to pesticides was linked with CKD in higher- and lower-income settings^46^. It can affect kidney function in combination with other occupational risk factors, such as heat stress, dehydration, intense workload, and exposure to chemicals, in disturbing kidney function. This is particularly, but not exclusively, true in agricultural settings^43,47^. In the US, pesticide applicators with long-term exposure to pesticides, including the herbicides alachlor, atrazine, metolachlor, paraquat and pendimethalin, and the insecticide permethrin, had higher risk of end-stage renal disease (ESRD). Other non-herbicide pesticides were also positively, but not significantly associated. Multiple medical visits due to pesticide exposure (hazard ratio – HR 2.13; 95% CI 1.17, 3.89) and hospitalization after pesticide use (HR 3.05; 95% CI 1.67, 5.58) were also significantly associated with ESRD^40^. Another study in the US also noted that occupational exposure to pesticides was related to increased ESRD risk (HR 1.78; 95% CI 1.36, 2.34)^48^.

A study with sugarcane farmers in Nicaragua found significant associations between lower eGFR and accidental pesticide inhalation history (OR 3.31, 95%CI 1.32–8.31)^49^. Farmworkers in Mexico working in conventional crops had lower eGFR levels than organic farmers^50^. Moreover, in Panama, CKDnt patients from a nephrology reference hospital were significantly younger and more engaged in agricultural or transportation work than traditional CKD patients, and had more renal atrophy and hyperuricemia as clinical markers of CKDnt^5^.

Few studies addressed kidney effects of occupational pesticide exposure in Brazil. A recent study shows that acute kidney failure (AKF) mortality is increasing in urban and rural regions, especially among agricultural workers from rural areas with greater pesticide consumption, younger workers, females, and those living in southern Brazil^51^. Another study with Brazilian farmers observed a lack of personal protective equipment (PPE) use and little occupational training, and found that workers with more than 5 years of pesticide exposure had higher relative risk of GFR alteration (RR 1.59)^52^.

Brazilian smallholder farmers are highly exposed to multiple pesticides as they handle and apply them without full PPE, perform unskilled labor on or near croplands, clean and store equipment and chemicals, live near croplands, and use pesticides in homes and home gardens. Most workers have low educational level and income, and lack occupational training or technical support, which compromise their ability to meet health and safety standards^12,53,54^.

Although there is a growing body of evidence on renal effects of pesticide exposure, some findings are still controversial^46,47^. A recent review shows that six out of nine studies conducted in Latin American countries reported null associations of pesticide with eGFR levels or prevalence of CKD^55^. Most of these studies had cross-sectional design, small sample size, and were based on self-reported estimation of exposure, which may result in information bias, imprecise exposure levels, limiting the study’s validity. Although some studies did not find a clear association between pesticide exposure and CKDnt, its influence as a strong contributor cannot be definitively ruled out without adequate exposure assessments^47^.

### Brazilian Regulation and Public Policies onWorkers’ Exposure to Heat, Metals, and Pesticides

Some Brazilian labor laws address workers’ exposure to heat, metals, and pesticides. They define procedures for identification and evaluation of occupational exposures to physical, chemical and biological agents, set workers’ exposure limits, including heat and metals (manganese, arsenic, chromium, lead, and mercury), define preventive and control measures for occupational exposures^56,57^, determine hazard pay for workers overexposed to harmful agents^57^, and stablish clinical follow-up and monitoring to identify workers’ metabolic and physiological alterations at an early stage^58^.

The quantitative assessment of occupational exposure to heat considers the thermal overload and worker’s metabolic rate by type of activity^56,59^. For agents lacking defined tolerance limits, prevention measures should consider those provided by the American Conference of Governmental Industrial Hygienists^56^. Furthermore, there are specific labor norms for occupational health and safety (OSH) in open air work^60^, construction^61^, mining^62^, agriculture, livestock, forestry, and aquaculture^63^, where workers are often exposed to heat, pesticides, and metals.

Concerning metals, Brazil is signatory of the Minamata Convention on Mercury and must implement actions to prevent and remediate mercury exposure. In this context, the Ministry of Health is conducting a an occupational exposure matrix to estimate the country’s exposed workers (Carex Brazil), but the results have not yet been published. In 2006, the Ministry of Health published a series of protocols for health services to facilitate the workers’ health surveillance, including a protocol about lead exposure. It is currently being reviewed and expanded to cover three metals, namely lead, chromium and mercury, but it has not yet been published. Technical publications on other metals are still lacking. Moreover, a population biomonitoring program is being developed by the Brazilian Ministry of Health, but it is still in its initial stage, and as of December, 2023, no biomarkers have yet been collected and analyzed.

The first Brazilian regulation on pesticides dates back to 1934, but it was only after 1989 that legal standards were set to regulate pesticide registration, use, production, storage, transport, and disposal through the Federal Law nº 7802/1989 and the Acts nº 4074/2002 and 5981/2006^64^. Moreover, agricultural labor regulation establishes criteria for OSH, presents potential risks, and sets protection guidelines, mandatory training, and prevention programs, such as the Risk Management Program in Rural Work (PGRTR), the Specialized Service in Safety and Health in Rural Work (SESTR), and the Internal Commission for the Prevention of Accidents in Rural Work (CIPATR)^63^. In these norms, organophosphate and organochlorine pesticides are recognized as hazardous substances subject to hazard pay^57^, although no other chemical classes are mentioned and no tolerance limits are defined. Regular training on pesticides and accident prevention is specified, but no regular biomonitoring programs for exposed workers are defined^63^.

Although labor regulation is quite comprehensive in Brazil, they are mandatory only for formally hired workers, and employers who do not follow these norms can be fined. Moreover, Brazil does not have sufficient infrastructure to oversee workers’ labor conditions in the whole country and depends mainly on formal complaints for law enforcement. Importantly, millions of informal and self-employed workers may be overexposed and can only rely on their self-awareness and behavior to protect themselves, as they are not covered by labor regulations and norms.

Regarding health actions, besides the National Policy for Workers’ Health (PNSTT) and the National Policy for Health Surveillance (PNVS), some policies and programs of the Ministry of Health are focused on the agricultural sector and pesticide surveillance, such as the 2012 Health Surveillance of Populations Exposed to Pesticides (VSPEA), which aims to implement integrated measures to prevent exposure and promote health surveillance of individuals exposed to pesticides, and the National Policy for the Comprehensive Health of the Rural, Forest and Water Populations (PNSIPCFA), created in 2011 to promote actions that help to prevent diseases and promote health^65^. Moreover, other initiatives were designed to promote sustainable rural development and pesticide restriction in Brazil, such as the National Program for Pesticide Reduction (PRONARA), and the National Policy for Agroecology and Organic Production (PNAPO). However, some of these initiatives were never fully implemented, and others were discontinued or weakened by the political changes under the Bolsonaro government (2019–2022)^66^.

Recently, Brazil published the new regulatory framework for pesticides, and adopted the Globally Harmonized System (GHS) of classification and labeling, updating the toxicological classification criteria, reducing the importance of chronic effects, reclassifying many products as less toxic, and changing the safety information on pesticide labels and packaging^67^. These changes were highly questioned by specialists, mainly because they reduced the acute risks and were not accompanied by risk communication and information campaigns to ensure that exposed workers understood these changes.

There are also regulations on chemical contamination of environmental matrices, such as soil and food. The National Council for the Environment (CONAMA) sets reference limits for metals in soil and guidelines for environmental management of contaminated areas^68^, while the National Health Surveillance Agency (Anvisa) provides the maximum tolerated limits (LMT) of metals and pesticides in food, defines the analysis methods for conformity assessment, and performs screening programs^69^. Still, metal ans pesticides contamination in agricultural soil, river sediments, and aquatic life has been commonly reported^11,70,71^. This environmental contamination and the associated risks for kidney disease are extremely important, but this is beyond the scope of this manuscript.

To the best of our knowledge, there are no other ongoing health surveillance and assistance programs or actions in Brazil by the Ministry of Health related to heat, metal, and pesticide exposures, nor are there any published documents, protocols, or guidelines focused on the effects of heat stress, metals, and pesticides on the kidneys.

## Conclusions

This descriptive review highlights the urgent need for more studies in Brazil, Latin American countries, and other lower-and-middle income countries to investigate the effects of environmental and occupational exposure to heat stress, metals and pesticides on kidney function, especially with longitudinal design, reliable biomarkers, and focusing on the most vulnerable workers^43,55^, such as rural workers. It is estimated that Brazil has more than 18 million rural workers, accounting for 20% of the country’s economically active population^72^. In general, they have low educational and income levels, and are more concentrated in less industrialized regions, with worse social and health indices^73^, where public health services and specialized services are not always available. Usually, rural populations do not seek medical care until symptoms and functional limitations are already advanced^73^. This is particularly worrisome for CKD detection, since it is a silent disease and symptoms only appear when kidney function is very low. Moreover, rural workers in Brazil are still commonly found in modern slavery work and some are rescued by justice agents in precarious sanitary conditions^74^.

The health and safety of workers in Brazil must be promoted and regulations, policies, and programs to protect workers (and the general population) from heat, metals, and pesticide exposures must be strengthened. Existing programs must be reinforced, and further programmatic actions, such as those related to the training and qualification of health professionals need to be designed and implemented to raise awareness about risk factors for CKDnt. Moreover, it is important to assess kidney effects of short- and long-term exposures, especially in the most exposed and vulnerable workers. Thus, establishing a comprehensive and coordinated national biomonitoring program can help to reveal the true extent of workers’ exposure and the impact on kidney health.

In this sense, regulatory, enforcement, and health surveillance initiatives are urgently needed, as is a partnership with society representatives, private companies, and academia, to investigate and implement strategies and mitigate the adverse health effects linked to exposure to heat stress, metals and pesticides.
